# The ChoCO-W prospective observational global study: Does COVID-19 increase gangrenous cholecystitis?

**DOI:** 10.1186/s13017-022-00466-4

**Published:** 2022-12-16

**Authors:** Belinda De Simone, Fikri M. Abu-Zidan, Elie Chouillard, Salomone Di Saverio, Massimo Sartelli, Mauro Podda, Carlos Augusto Gomes, Ernest E. Moore, Susan J. Moug, Luca Ansaloni, Yoram Kluger, Federico Coccolini, Aitor Landaluce-Olavarria, Begoña Estraviz-Mateos, Ana Uriguen-Etxeberria, Alessio Giordano, Alfonso Palmieri Luna, Luz Adriana Hernández Amín, Adriana María Palmieri Hernández, Amanda Shabana, Zakaria Andee Dzulkarnaen, Muhammad Asyraf Othman, Mohamad Ikhwan Sani, Andrea Balla, Rosa Scaramuzzo, Pasquale Lepiane, Andrea Bottari, Fabio Staderini, Fabio Cianchi, Andrea Cavallaro, Antonio Zanghì, Alessandro Cappellani, Roberto Campagnacci, Angela Maurizi, Mario Martinotti, Annamaria Ruggieri, Asri Che Jusoh, Karim Abdul Rahman, Anis Suraya M. Zulkifli, Barbara Petronio, Belén Matías-García, Ana Quiroga-Valcárcel, Fernando Mendoza-Moreno, Boyko Atanasov, Fabio Cesare Campanile, Ilaria Vecchioni, Luca Cardinali, Grazia Travaglini, Elisa Sebastiani, Serge Chooklin, Serhii Chuklin, Pasquale Cianci, Enrico Restini, Sabino Capuzzolo, Giuseppe Currò, Rosalinda Filippo, Michele Rispoli, Daniel Aparicio-Sánchez, Virginia Durán Muñóz-Cruzado, Sandra Dios Barbeito, Samir Delibegovic, Amar Kesetovic, Diego Sasia, Felice Borghi, Giorgio Giraudo, Diego Visconti, Emanuele Doria, Mauro Santarelli, Davide Luppi, Stefano Bonilauri, Ugo Grossi, Giacomo Zanus, Alberto Sartori, Giacomo Piatto, Maurizio De Luca, Domenico Vita, Luigi Conti, Patrizio Capelli, Gaetano Maria Cattaneo, Athanasios Marinis, Styliani-Aikaterini Vederaki, Mehmet Bayrak, Yasemin Altıntas, Mustafa Yener Uzunoglu, Iskender Eren Demirbas, Yuksel Altinel, Serhat Meric, Yunus Emre Aktimur, Derya Salim Uymaz, Nail Omarov, Ibrahim Azamat, Eftychios Lostoridis, Eleni-Aikaterini Nagorni, Antonio Pujante, Gabriele Anania, Cristina Bombardini, Francesco Bagolini, Emre Gonullu, Baris Mantoglu, Recayi Capoglu, Stefano Cappato, Elena Muzio, Elif Colak, Suleyman Polat, Zehra Alan Koylu, Fatih Altintoprak, Zülfü Bayhan, Emrah Akin, Enrico Andolfi, Sulce Rezart, Jae Il Kim, Sung Won Jung, Yong Chan Shin, Octavian Enciu, Elena Adelina Toma, Fabio Medas, Gian Luigi Canu, Federico Cappellacci, Fabrizio D’Acapito, Giorgio Ercolani, Leonardo Solaini, Francesco Roscio, Federico Clerici, Roberta Gelmini, Francesco Serra, Elena Giulia Rossi, Francesco Fleres, Guglielmo Clarizia, Alessandro Spolini, Francesco Ferrara, Gabriela Nita, Jlenia Sarnari, Mahir Gachabayov, Abakar Abdullaev, Gaetano Poillucci, Gian Marco Palini, Simone Veneroni, Gianluca Garulli, Micaela Piccoli, Gianmaria Casoni Pattacini, Francesca Pecchini, Giulio Argenio, Mariano Fortunato Armellino, Giuseppe Brisinda, Silvia Tedesco, Pietro Fransvea, Giuseppe Ietto, Caterina Franchi, Giulio Carcano, Gennaro Martines, Giuseppe Trigiante, Giulia Negro, Gustavo Machain Vega, Agustín Rodríguez González, Leonardo Ojeda, Gaetano Piccolo, Andrea Bondurri, Anna Maffioli, Claudio Guerci, Boo Han Sin, Zamri Zuhdi, Azlanudin Azman, Hussam Mousa, Shadi al Bahri, Goran Augustin, Ivan Romic, Trpimir Moric, Ioannis Nikolopoulos, Jacopo Andreuccetti, Giusto Pignata, Rossella D’Alessio, Jakub Kenig, Urszula Skorus, Gustavo Pereira Fraga, Elcio Shiyoiti Hirano, Jackson Vinícius de Lima Bertuol, Arda Isik, Eray Kurnaz, Mohammad Sohail Asghar, Ameer Afzal, Ali Akbar, Taxiarchis Konstantinos Nikolouzakis, Konstantinos Lasithiotakis, Emmanuel Chrysos, Koray Das, Nazmi Özer, Ahmet Seker, Mohamed Ibrahim, Hytham K. S. Hamid, Ahmed Babiker, Konstantinos Bouliaris, George Koukoulis, Chrysoula-Christina Kolla, Andrea Lucchi, Laura Agostinelli, Antonio Taddei, Laura Fortuna, Carlotta Agostini, Leo Licari, Simona Viola, Cosimo Callari, Letizia Laface, Emmanuele Abate, Massimiliano Casati, Alessandro Anastasi, Giuseppe Canonico, Linda Gabellini, Lorenzo Tosi, Anna Guariniello, Federico Zanzi, Lovenish Bains, Larysa Sydorchuk, Oksana Iftoda, Andrii Sydorchuk, Michele Malerba, Federico Costanzo, Raffaele Galleano, Michela Monteleone, Andrea Costanzi, Carlo Riva, Maciej Walędziak, Andrzej Kwiatkowski, Łukasz Czyżykowski, Piotr Major, Marcin Strzałka, Maciej Matyja, Michal Natkaniec, Maria Rosaria Valenti, Maria Domenica Pia Di Vita, Maria Sotiropoulou, Stylianos Kapiris, Damien Massalou, Massimiliano Veroux, Alessio Volpicelli, Rossella Gioco, Matteo Uccelli, Marta Bonaldi, Stefano Olmi, Matteo Nardi, Giada Livadoti, Cristian Mesina, Theodor Viorel Dumitrescu, Mihai Calin Ciorbagiu, Michele Ammendola, Giorgio Ammerata, Roberto Romano, Mihail Slavchev, Evangelos P. Misiakos, Emmanouil Pikoulis, Dimitrios Papaconstantinou, Mohamed Elbahnasawy, Sherief Abdel-elsalam, Daniel M. Felsenreich, Julia Jedamzik, Nikolaos V. Michalopoulos, Theodoros A. Sidiropoulos, Maria Papadoliopoulou, Nicola Cillara, Antonello Deserra, Alessandro Cannavera, Ionuţ Negoi, Dimitrios Schizas, Athanasios Syllaios, Ilias Vagios, Stavros Gourgiotis, Nick Dai, Rekha Gurung, Marcus Norrey, Antonio Pesce, Carlo Vittorio Feo, Nicolo’ Fabbri, Nikolaos Machairas, Panagiotis Dorovinis, Myrto D. Keramida, Francesk Mulita, Georgios Ioannis Verras, Michail Vailas, Omer Yalkin, Nidal Iflazoglu, Direnc Yigit, Oussama Baraket, Karim Ayed, Mohamed hedi Ghalloussi, Parmenion Patias, Georgios Ntokos, Razrim Rahim, Miklosh Bala, Asaf Kedar, Robert G. Sawyer, Anna Trinh, Kelsey Miller, Ruslan Sydorchuk, Ruslan Knut, Oleksandr Plehutsa, Rumeysa Kevser Liman, Zeynep Ozkan, Saleh Abdel Kader, Sanjay Gupta, Monika Gureh, Sara Saeidi, Mohsen Aliakbarian, Amin Dalili, Tomohisa Shoko, Mitsuaki Kojima, Raira Nakamoto, Semra Demirli Atici, Gizem Kilinc Tuncer, Tayfun Kaya, Spiros G. Delis, Stefano Rossi, Biagio Picardi, Simone Rossi del Monte, Tania Triantafyllou, Dimitrios Theodorou, Tadeja Pintar, Jure Salobir, Dimitrios K. Manatakis, Nikolaos Tasis, Vasileios Acheimastos, Orestis Ioannidis, Lydia Loutzidou, Savvas Symeonidis, Tiago Correia de Sá, Mónica Rocha, Tommaso Guagni, Desiré Pantalone, Gherardo Maltinti, Vladimir Khokha, Wafaa Abdel-elsalam, Basma Ghoneim, José Antonio López-Ruiz, Yasin Kara, Syaza Zainudin, Firdaus Hayati, Nornazirah Azizan, Victoria Tan Phooi Khei, Rebecca Choy Xin Yi, Harivinthan Sellappan, Zaza Demetrashvili, Nika Lekiashvili, Ana Tvaladze, Caterina Froiio, Daniele Bernardi, Luigi Bonavina, Angeles Gil-Olarte, Sebastiano Grassia, Estela Romero-Vargas, Francesco Bianco, Andrew A. Gumbs, Agron Dogjani, Ferdinando Agresta, Andrey Litvin, Zsolt J. Balogh, George Gendrikson, Costanza Martino, Dimitrios Damaskos, Nikolaos Pararas, Andrew Kirkpatrick, Mikhail Kurtenkov, Felipe Couto Gomes, Adolfo Pisanu, Oreste Nardello, Fabrizio Gambarini, Hager Aref, Nicola de’ Angelis, Vanni Agnoletti, Antonio Biondi, Marco Vacante, Giulia Griggio, Roberta Tutino, Marco Massani, Giovanni Bisetto, Savino Occhionorelli, Dario Andreotti, Domenico Lacavalla, Walter L. Biffl, Fausto Catena

**Affiliations:** 1 Department of Emergency, Digestive and Metabolic Minimally Invasive Surgery, Poissy and Saint Germain en Laye Hospitals, Poissy, France; 2 The Research Office, College of Medicine and Health Sciences, United Arab Emirates University, Al-Ain, United Arab Emirates., United Arab Emirates University, Al-Ain, UAE; 3 Department of General Surgery, Santa Maria del Soccorso Hospital, San Benedetto del Tronto, Ascoli Piceno, Italy; 4 Department of General Surgery, Macerata Hospital, Macerata, Italy; 5 Department of Surgical Science, University of Cagliari, Cagliari, Italy; 6 Faculdade de Ciência Médicas e da Saúde de Juiz de Fora, Hospital Universitario Terezinha de Jesus (SUPREMA), Juiz de Fora, Brazil; 7 Ernest E. Moore Shock Trauma Center at Denver Health, Denver, CO USA; 8 Department of Surgery, Royal Alexandra Hospital, Paisley and Golden Jubilee National Hospital, University of Glasgow, Glasgow, Scotland; 9 Department of General Surgery, University Hospital of Pavia, Pavia, Italy; 10 Department of General Surgery, The Rambam Academic Hospital, Haifa, Israel; 11 Department of General and Emergency Surgery, University Hospital of Pisa, Pisa, Italy; 12 Alfredo- Espinosa Urduliz Hospital, Urduliz, Spain; 13 General Surgery Unit ASL Toscana Centro, Santo Stefano Hospital, Prato, Italy; 14 Universidad de Sucre, Clínica Santa María, Sincelejo, Colombia; 15 Emergency Surgery Department, John Radcliffe Hospital, Oxford, UK; 16 Department of Surgery, School of Medical Sciences and Hospital USM, Universiti Sains Malaysia, Kubang Kerian, Kelantan Malaysia; 17 UOC of General and Minimally Invasive Surgery, Hospital “San Paolo”, Largo Donatori del Sangue 1, 00053 Civitavecchia, Rome, Italy; 18 SOD Chirurgia dell’Apparato Digerente, AOU Careggi, Florence, Italy; 19 Department of Surgery, University of Catania, Policlinico “G. Rodolico - San Marco” Hospital, Catania, Italy; 20 U.O.C. of General Surgery, “Carlo Urbani” Hospital, Jesi, AN Italy; 21 Hospital “Istituto Città di Pavia”, Pavia, Italy; 22 Department of General Surgery, Hospital Sultan Ismail Petra, 18000 Kuala Krai, Kelantan Malaysia; 23 Chirurgia Generale e Mininvasiva, San Polo Monfalcone, Monfalcone, GO Italy; 24 Hospital Universitario Príncipe de Asturias, Alcalá de Henares, Spain; 25 RIMU, Medical University of Plovdiv, UMHAT Eurohospital, Plovdiv, Bulgaria; 26 Ospedale San Giovanni Decollato Andosilla – ASL, Civita Castellana, Viterbo, VT Italy; 27 UOC Chirurgia Generale, Ospedale “Madonna del Soccorso”, San Benedetto del Tronto, AP Italy; 28 Lviv Regional Clinical Hospital, Lviv, Ukraine; 29 Lorenzo Bonomo Hospital, ASL BAT, Andria, Puglia Italy; 30 Generall Surgery Unit, Science of Health Department, “Mater Domini” Hospital, University “Magna Graecia” Medical School, Viale Europa, 88100 Germaneto, Catanzaro Italy; 31 Emergency Surgery Unit, Hospital Virgen del Rocío, Seville, Spain; 32 Clinic for Surgery, University Clinical Center Tuzla, Tuzla, Bosnia and Herzegovina; 33 Santa Croce and Carle Hospital, Cuneo, Italy; 34 Chirurgia Generale d’Urgenza e PS - AOU Cittá della Salute e della Scienza, Turin, Italy; 35 General and Emergency Surgery, ASMN IRCCS REGGIO EMILIA, Reggio Emilia, Italy; 36 Surgery Unit 2, Regional Hospital Treviso, DISCOG, University of Padua, Treviso, Italy; 37 U.O. Chirurgia Generale e d’Urgenza Ospedale San Valentino, Montebelluna, Treviso, Italy; 38 Department of Surgery, G. Da Saliceto Hospital, AUSL Piacenza, Piacenza, Italy; 39 Styliani-Aikaterini Vederaki, Third Department of Surgery, “Tzaneio” General Hospital, Piraeus, Greece; 40 Mehmet Bayrak, Clinic for Surgery, Private Ortadogu Hospital, Adana, Turkey; 41 Clinic for Radiology, Private Medline Hospital, Adana, Turkey; 42 Department of General Surgery, Kestel State Hospital, Bursa, Turkey; 43 Department of General Surgery, Bagcilar Training and Research Hospital, University of Health Science, Istanbul, Turkey; 44 General Surgery Department, Faculty of Medicine, Koc University, Istanbul, Turkey; 45 1St Department of Surgery, Kavala General Hospital, Kavala, Greece; 46 UO Chirurgia 1, Dipartimento Chirurgico, Arcispedale Sant’Anna, Azienda Ospedaliero-Universitaria’di Ferrara, Ferrara, Italy; 47 Sakarya Training and Research Hospital, Sakarya, Turkey; 48 Department of General Surgery ASL 4, Lavagna Hospital, Genoa, Italy; 49 Samsun Training and Research Hospital, University of Samsun, Samsun, Turkey; 50 Department of General Surgery, Faculty of Medicine, Sakarya University, Serdivan, Turkey; 51 General and Emergency Surgery Unit, San Donato Hospital, Arezzo, Italy; 52 Department of Surgery, Inje University Ilsan Paik Hospital, Goyang, South Korea; 53 Elias University Emergency Hospital, Carol Davila University of Medicine and Pharmacy, Bucharest, Romania; 54 General and Oncologic Surgery, Morgagni-Pierantoni Hospital, AUSL Romagna, Via C. Forlanini 34, 47121 Forlì, Italy; 55 Emergency Surgery, ASST Valle Olona, Busto Arsizio, Italy; 56 Department of Medical and Surgical Sciences for Children and Adults, University of Modena and Reggio Emilia School of Medicine AOU Policlinico Di Modena, Modena, Italy; 57 UOC General Surgery, Hospital Civil of Sondrio, ASST Valtellina e Alto Lario, Sondrio, Italy; 58 Department of Surgery, San Carlo Borromeo Hospital, ASST Santi Paolo e Carlo, Milan, Italy; 59 AUSL Reggio Emilia, Ospedale Sant’Anna, Castelnuovo ne Monti, Reggio Emilia, Italy; 60 Department of Abdominal Surgery, Vladimir City Emergency Hospital, Vladimir, Russia; 61 Policlinico Universitario Umberto I, Rome, Italy; 62 Chirurgia generale e d’urgenza, Ospedale Infermi di Rimini, AUSL Romagna, Rimini, Italy; 63 Department of General Surgery, Emergencies and New Technologies, Baggiovara Civil Hospital, Modena, Italy; 64 UOC Chirurgia d’Urgenza, AOU San Giovanni di Dio e Ruggi d’Aragona, Salerno, Italy; 65 Department of Medical and Surgical Sciences, Fondazione Policlinico Universitario A Gemelli IRCCS, Rome, Italy; 66 General, Emergency and Transplant Surgery Department, ASST-Settelaghi and University of Insubria, Varese, Italy; 67 General Surgery Unit, Azienda Ospedaliero Universitaria Policlinico Bari - Italy, Bari, Italy; 68 Department of Surgery, Hospital de Clinicas, Universidad Nacional de Asunción, San Lorenzo, Paraguay; 69 Unit of HepatoBilioPancreatic and Digestive Surgery, Department of Health Sciences, San Paolo Hospital, University of Milan, Via Di Rudinì 8, 20142 Milan, Italy; 70 Department of General Surgery, Department of Biomedical and Clinical Sciences Luigi Sacco, Luigi Sacco University Hospital, Università degli Studi di Milano, Milan, Italy; 71 HPB Unit, Department of Surgery, Hospital Canselor Tuanku Muhriz, Kuala Lumpur, Malaysia; 72 College of Medicine, Tawam Hospital, UAE University, Al-Ain, UAE; 73 Department of Surgery, University Hospital Centre, Zagreb, Croatia; 74 Lewisham and Greenwich NHS Trust, London, UK; 75 2nd Department of General Surgery, ASST Spedali Civili of Brescia, Brescia, Italy; 76 Department of General, Gastrointestinal, Oncologic Surgery and Transplantology, Jagiellonian University Medical College, Kraków, Poland; 77 Division of Trauma Surgery, School of Medical Sciences, University of Campinas (Unicamp), Campinas, Brazil; 78 Division of General Surgery, Western Paraná University Hospital (Huop-Unioeste), Cascavel, Brazil; 79 School of Medicine, Erzincan University, Erzincan, Turkey; 80 King Edward Medical University, Lahore, Pakistan; 81 Department of General Surgery, University General Hospital of Heraklion, 71110 Heraklion, Crete, Greece; 82 Department of General Surgery, Adana City Training and Research Hospital, University of Health Sciences, Adana, Turkey; 83 Kuwaiti Specialized Hospital, Khartoum, Sudan; 84 Surgical Department, Koutlimbaneio and Triantafylleio General Hospital of Larissa, Larisa, Greece; 85 U.O. Chirurgia Generale Ospedale “Ceccarini” Riccione, Riccione, Italy; 86 Hepatobiliary Surgery, Department of Clinical and Experimental Medicine, University of Florence, AOU Careggi, Florence, Italy; 87 Department of Surgical, Oncological and Oral Sciences (DICHIRONS), Policlinico P. Giaccone, University of Palermo, Via Liborio Giuffré 5, 90127 Palermo, Italy; 88 University of Palermo, Palermo, Italy; 89 Department of Surgery, Buccheri La Ferla Hospital, Via Messina Marine, 197, 90123 Palermo, Italy; 90 Department of General Surgery, Vittorio Emanuele III Hospital, Carate Brianza - ASST Brianza, Carate Brianza, Italy; 91 Chirurgia Generale, Ospedale San Giovanni Di Dio, Florence, Italy; 92 Section of Emergency Surgery, Department of Surgery, S.Maria delle Croci Hospital Ravenna, Ravenna, Italy; 93 Residency Program in General Surgery, University of Bologna, Bologna, Italy; 94 Department of Surgery, Maulana Azad Medical College and Nayak Hospital, New Delhi, 110002 India; 95 Bukovinian State Medical University, Chernivtsi, Ukraine; 96 Ospedale Santa Corona, ASL 2, Savona, Italy; 97 Andrea Costanzi, Carlo Riva, O.U. of General Surgery, San Leopoldo Mandic Hospital, Merate, ASST, Lecco, Italy; 98 Department of General, Oncological, Metabolic and Thoracic Surgery, Military Institute of Medicine, Warsaw, Poland; 99 Department of General and Emergency Surgery, Faculty of Medicine, Jagiellonian University Medical College, Kraków, Poland; 100 U.O. General Surgery, Azienda Ospedaliera Universitaria “Policlinico - San Marco”, Catania, Italy; 101 3Rd Surgical Department, Evangelismos General Hospital, Athens, Greece; 102 Department of Emergency Surgery, Centre Hospitalier Universitaire de Nice (CHU de Nice), Université Côte d’Azur, Nice, France; 103 General Surgery, Azienda Policlinico San Marco, Catania, Italy; 104 General and Oncological Surgery Department, San Marco Hospital GSD, Zingonia, BG Italy; 105 San Giovanni Calibita Hospital- Fondazione Fatebenefratelli, Rome, Italy; 106 Department of Surgery, Emergency County Hospital of Craiova, Craiova, Romania; 107 Science of Health Department, Digestive Surgery Unit, “Mater Domini” Hospital, University “Magna Graecia” Medical School, Viale Europa, 88100 Germaneto, Catanzaro Italy; 108 Department of General Surgery, University Hospital Eurohospital, Plovdiv, Bulgaria; 109 3Rd Department of Surgery, Attikon University Hospital, National and Kapodistrian University of Athens, Athens, Greece; 110 Emergency Medicine and Traumatology Department, Tanta University Faculty of Medicine, Tanta, Egypt; 111 Tropical Medicine and Infectious Diseases, Faculty of Medicine, Tanta University, Tanta, Egypt; 112 Division of Visceral Surgery, Department of General Surgery, Medical University of Vienna, Vienna, Austria; 113 4Rd Department of Surgery Attikon University Hospital, National and Kapodistrian University of Athens, Athens, Greece; 114 Surgery Department, Santissima Trinità Hospital, Cagliari, Italy; 115 General Surgery Department, Carol Davila University of Medicine and Pharmacy, Emergency Hospital of Bucharest, Bucharest, Romania; 116 First Department of Surgery, National and Kapodistrian University of Athens, Laiko General Hospital, 11527 Athens, Greece; 117 Addenbrooke’s Hospital, Cambridge University, Cambridge, UK; 118 Department of Surgery, Delta Hospital, Azienda USL of Ferrara, University of Ferrara, Ferrara, Italy; 119 2Nd Department of Propaedeutic Surgery, National and Kapodistrian University of Athens, General Hospital Laiko, Athens, Greece; 120 Department of Surgery, General University Hospital of Patras, Patras, Greece; 121 Department of Surgical Oncology and Gastroenterological Surgery, Bursa City Hospital, Bursa, Turkey; 122 Department of General Surgery, Habib Bougatfa Hospital, University Tunis El Manar, Bizerte, Tunisia; 123 2nd Department of Surgery, General Hospital of Athens “G.Gennimatas”, Athens, Greece; 124 Department of Surgery, Universiti Sains Islam Malaysia, Nilai, Malaysia; 125 Department of General Surgery and Trauma, Hadassah Medical Center and Faculty of Medicine, Hebrew University of Jerusalem, Jerusalem, Israel; 126 Western Michigan University School of Medicine, Kalamazoo, USA; 127 Regional Emergency Hospital, Chernivtsi, Ukraine; 128 General Surgery Clinic, Elazig Fethi Sekin City Hospital, Elazig, Turkey; 129 Egypt and NMC Specialty Hospital Al Ain, Ain Shams University, Al-Ain, UAE; 130 Government Medical College and Hospital, Chandigarh, India; 131 Surgical Oncology Research Center, Mashhad University of Medical Sciences, Mashhad, Iran; 132 Department of Emergency and Critical Care Medicine, Department of Acute Care Surgery Center, Adachi Medical Center, Tokyo Women’s Medical University, Tokyo, Japan; 133 Department of General Surgery, University of Health Sciences Tepecik Training and Research Hospital, Izmir, Turkey; 134 HPB Unit Konstantopouleio Hospital St Olga, Athens, Greece; 135 Department of General and Emergency Surgery, San Filippo Neri Hospital, ASL Roma 1, Rome, Italy; 136 Department of Surgery, Hippocration General Hospital of Athens, University of Athens, Athens, Greece; 137 Department of Abdominal Surgery, University Medical Center Ljubljana, Ljubljana, Slovenia; 138 Vasileios Acheimastos, Athens Naval and Veterans Hospital, Athens, Greece; 139 4Th Department of Surgery, Medical School Aristotle University of Thessaloniki, General Hospital “George Papanikolaou”, Thessaloniki, Greece; 140 General Surgery Department, Centro Hospitalar Do Tâmega e Sousa Penafiel, Penafiel, Portugal; 141 Department of general surgery, Careggi University Hospital, Florence, Italy; 142 Emergency Surgery Department, City Hospital, Mozyr, Belarus; 143 Anesthesia and Surgical Intensive Care Department, Faculty of Medicine, Kafrelsheikh University, Kafrelsheikh, Egypt; 144 Angeles Gil-Olarte, Estela Romero-Vargas, Hospital Universitario Virgen Macarena, Seville, Spain; 145 General Surgery Clinic Health Sciences University Kanuni Sultan Süleyman Training and Research Hospital, Istanbul, Turkey; 146 Faculty of Medicine and Health Sciences, Queen Elisabeth Hospital, Universiti Malaysia Sabah, Kota Kinabalu, Malaysia; 147 Department of Surgery, Queen Elizabeth Hospital, Universiti Malaysia Sabah, Kota Kinabalu, Malaysia; 148 N.Kipshidze Central University Hospital, Tbilisi, Georgia; 149 IRCCS Policlinico San Donato, University of Milan, Milan, Italy; 150 General Surgery Unit, S. Leonardo Hospital, Castellammare Di Stabia, Naples, Italy; 151 Department of General Surgery, University Hospital of Tirana, Tirana, Albania; 152 Department of General Surgery, AULSS2 Trevigiana del Veneto, Ospedale di Vittorio Veneto, Vittorio Veneto, TV Italy; 153 Department of Surgical Disciplines, Immanuel Kant Baltic Federal University, Regional Clinical Hospital, Kalingrad, Russia; 154 Department of Traumatology, John Hunter Hospital and University of Newcastle, Newcastle, NSW Australia; 155 Anesthesia and Intensive Care Unit, Umberto I Hospital, AUSL Romagna, Lugo, Italy; 156 Department of General and Emergency Surgery, Royal Infirmary of Edinburgh, University of Edinburgh, Edinburgh, UK; 157 Department of General Surgery, Dr. Sulaiman Al Habib Hospital, Alfaisal University, Riyadh, Saudi Arabia; 158 General, Acute Care, Abdominal Wall Reconstruction, and Trauma Surgery, Foothills Medical Centre, Calgary, AB Canada; 159 Unit of Digestive and HPB Surgery, CARE Department, Henri Mondor Hospital and University Paris-Est, Creteil, France; 160 Department of General and Trauma Surgery, Bufalini Hospital, Cesena, Italy; 161 Department of Emergency and Trauma Surgery, Scripps Clinic Medical Group, La Jolla, CA USA; 162 Department of General Surgery and Medical-Surgical Specialties, University of Catania, Catania, Italy; 163 Chirurgia 1; Dipartimento di Discipline Chirurgiche , Oncologiche e Stomatologiche (DI.CHIR.ON.S), Ospedale “Ca’Foncell”; Univerità degli studi di Palermo, Treviso; Palermo, Italy; 164 Dipartimento di Scienze Chirurgiche, Oncologiche e Gastroenterologica (DI.SC.O.G.), Chirurgia 1-Ospedale “Ca Foncello”- Treviso, Università degli Studi di Padova, Padua, Italy; 165 Department of General Surgery, Arcispedale Sant’Anna-University of Ferrara, Ferrara, Italy

**Keywords:** Acute cholecystitis, Cholecystectomy, Gangrene, COVID-19, SARS-CoV-2, Laparoscopy, Surgery, Pandemic, Gangrenous cholecystitis

## Abstract

**Background:**

The incidence of the highly morbid and potentially lethal gangrenous cholecystitis was reportedly increased during the COVID-19 pandemic. The aim of the ChoCO-W study was to compare the clinical findings and outcomes of acute cholecystitis in patients who had COVID-19 disease with those who did not.

**Methods:**

Data were prospectively collected over 6 months (October 1, 2020, to April 30, 2021) with 1-month follow-up. In October 2020, Delta variant of SARS CoV-2 was isolated for the first time. Demographic and clinical data were analyzed and reported according to the STROBE guidelines. Baseline characteristics and clinical outcomes of patients who had COVID-19 were compared with those who did not.

**Results:**

A total of 2893 patients, from 42 countries, 218 centers, involved, with a median age of 61.3 (SD: 17.39) years were prospectively enrolled in this study; 1481 (51%) patients were males. One hundred and eighty (6.9%) patients were COVID-19 positive, while 2412 (93.1%) were negative. Concomitant preexisting diseases including cardiovascular diseases (*p* < 0.0001), diabetes (*p* < 0.0001), and severe chronic obstructive airway disease (*p* = 0.005) were significantly more frequent in the COVID-19 group. Markers of sepsis severity including ARDS (*p* < 0.0001), PIPAS score (*p* < 0.0001), WSES sepsis score (*p* < 0.0001), qSOFA (*p* < 0.0001), and Tokyo classification of severity of acute cholecystitis (*p* < 0.0001) were significantly higher in the COVID-19 group. The COVID-19 group had significantly higher postoperative complications (32.2% compared with 11.7%, *p* < 0.0001), longer mean hospital stay (13.21 compared with 6.51 days, *p* < 0.0001), and mortality rate (13.4% compared with 1.7%, *p* < 0.0001). The incidence of gangrenous cholecystitis was doubled in the COVID-19 group (40.7% compared with 22.3%). The mean wall thickness of the gallbladder was significantly higher in the COVID-19 group [6.32 (SD: 2.44) mm compared with 5.4 (SD: 3.45) mm; *p* < 0.0001].

**Conclusions:**

The incidence of gangrenous cholecystitis is higher in COVID patients compared with non-COVID patients admitted to the emergency department with acute cholecystitis. Gangrenous cholecystitis in COVID patients is associated with high-grade Clavien-Dindo postoperative complications, longer hospital stay and higher mortality rate. The open cholecystectomy rate is higher in COVID compared with non -COVID patients. It is recommended to delay the surgical treatment in COVID patients, when it is possible, to decrease morbidity and mortality rates. COVID-19 infection and gangrenous cholecystistis are not absolute contraindications to perform laparoscopic cholecystectomy, in a case by case evaluation, in expert hands.

**Graphical abstract:**

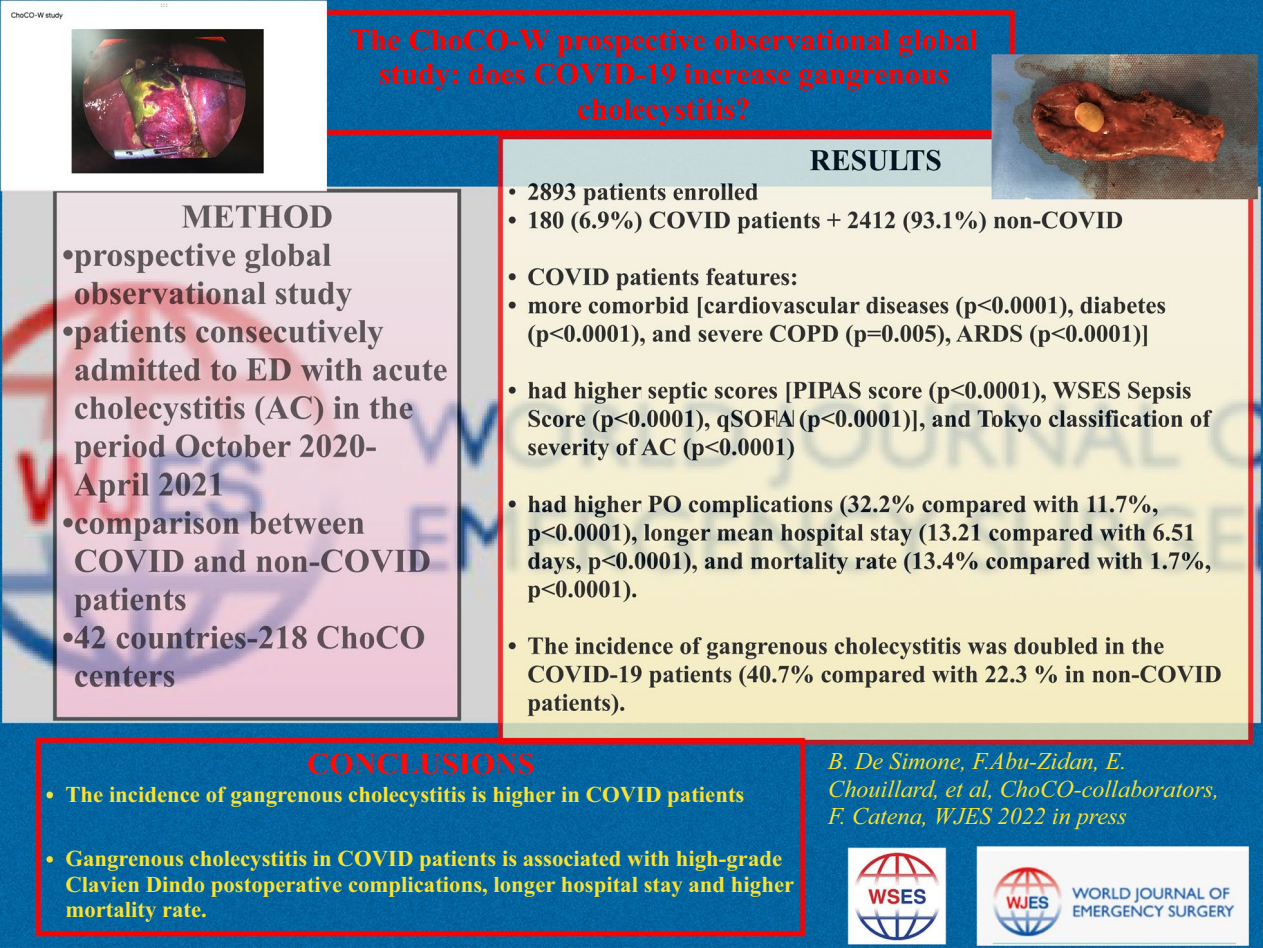

## Introduction

Acute cholecystitis (AC) is a common cause of emergency hospital admission that should be managed according to international guidelines [1, 2]. It can be classified into 3 grades of severity (mild, moderate, and severe). These grades affect the length of hospital stay, conversion to open surgery, medical costs, and prognosis [1]. Gangrenous cholecystitis (GC) is a severe form of AC. It occurs in approximately 15% of the patients (range 2–30%) and is associated with an increased risk of postoperative morbidity and mortality [3, 4]. During the COVID-19 pandemic, we observed an increased number of AC patients who presented with gangrenous acute cholecystitis. An early case series showed that COVID-19 infection and pneumonia were associated with GC with increased morbidity and mortality, mainly in elderly and frail patients [5–9].

GC requires prompt surgical management to reduce hospital stay and improve the clinical outcome. Several retrospective studies focused on the management of AC patients in the first period of COVID-19 pandemic. They reported increased non-operative management (NOM) in those patients. This was associated with increased conservative management failure, morbidity, and length of hospital stay (LOS). This was attributed to the limited access to the operating theaters in attempt to reduce the in-hospital spreading of the virus. Age, COVID-19 infection, AC severity, and NOM failure contributed to the increased death rate [10]. The aim of the ChoCO-W global prospective study is to compare the clinical course, biological and radiological findings, and clinical outcome of AC in patients who have COVID-19 disease with those who do not have it.

## Patients and methods

### Ethical considerations

Ethical committee approval was obtained from the CPP Sud-Méditerranée 3, University Hospital of Nîmes-France (2021.03.05 ter _ 21.01.16.09406). The ChoCO-W prospective study met and followed the standards outlined in the World Medical Association Declaration of Helsinki [11]. It did not change or modify the usual clinical practices of the participating acute care surgeons.

### Study protocol

The ChoCO-W study was registered in the ClinicalTrials.gov (ID: NCT04542312). The details of the protocol were published [12]. This study was conceived and designed to run over 12 months (October 2020–October 2021). It is a global collaborative, prospective cohort study, including consecutive adult patients admitted to emergency departments with AC who were screened for SARS-CoV-2 using quantitative reverse transcription polymerase chain reaction (RT-PCR) swab test. The recruitment period was for 6 months (October 1, 2020, to April 30, 2021) with 1 month of postoperative follow-up. Two hundred and eighteen ChoCO collaborating centers joined the project and participated in the study. Each international center constituted a ChoCO team (1 local investigator and 2 collaborators) which was linked to an ID number for entering data anonymously in a secured web database. All local investigators were responsible of patients recruitment, data collection, and research ethical issues according to their local standards. All ChoCO collaborators who collected and entered the data were included in the ChoCO-collaborative authorship. The prospectively collected data were reported according to the STROBE guidelines [13].

### Patients

A total of 2893, with a mean age of 61.3 years (SD 17.3), were prospectively included in the study. A total of 1481 (51%) patients were male. Three hundred and one patients did not have RT-PCR swab test for COVID-19 infection, or their results were non-conclusive, and they were excluded from the analysis. Out of the remaining 2592 patients with known PCR test result, 180 (6.9%) were proven to be COVID-19 positive and 2412 (93.1%) were COVID-19 negative. These two groups were compared. Concerning SARS-CoV-2 type, multiple variants emerged in the fall of 2020 and the most circulating in the recruitment period of the ChoCO-W study was the Delta variant (B.1.617.2), isolated firstly in India in October 2020. This variant showed higher virulence compared with wild-type SARS-CoV-2 [https://www.who.int/activities/tracking-SARS-CoV-2-variants#cms].

### Study variables

Demography, clinical, laboratory, radiological, surgical, microbiological, and histopathological data were prospectively collected. These included gender, age, details of clinical presentation, preoperative diagnosis, radiological workup, markers of inflammation, surgical procedures, critical care support, complications, need for surgery, histopathological findings, hospital stay, and clinical outcomes. Clinical severity of the disease was assessed with the qSOFA score [14], PIPAS severity score [15], WSES sepsis severity score [16], while the severity of AC was assessed with the Tokyo severity classification [1]. Postoperative complications were reported according to the Clavien-Dindo classification [17].

### Statistical analysis

Data were downloaded from the web database to Microsoft Excel (Microsoft Office 365, USA). Data were imported to an SPSS program, sorted, cleaned, and recoded as numbers. Missing data were not imputed, and the analysis was performed on all available data.

Patients were divided into 2 groups according to COVID-19 infection: non-COVID group and COVID group.

Data are presented as number (%) for categorical data, median (range) for ordinal data, and mean (SD) for continuous data. Data were presented as both median (range) and mean (SD) when there was statistically significant difference in the ranks which did not show in the median (range) numbers. This was meant for clarification as some may not appreciate the significant difference between the two groups despite having the same median (range). The reported valid percentages were calculated from the available data and not as percentage of the study population.

Nonparametric methods were used for the analysis as they are more protective and demanding than parametric methods; moreover, nonparametric methods can be used for small numbers and do not need a normal distribution. Fisher’s exact test was used to compare categorical data of independent groups, while Mann–Whitney U test was used to compare the ordinal or continuous data of two independent groups. A *p* value of less than 0.05 was accepted as significant.

## Results

**Fig. 1:**
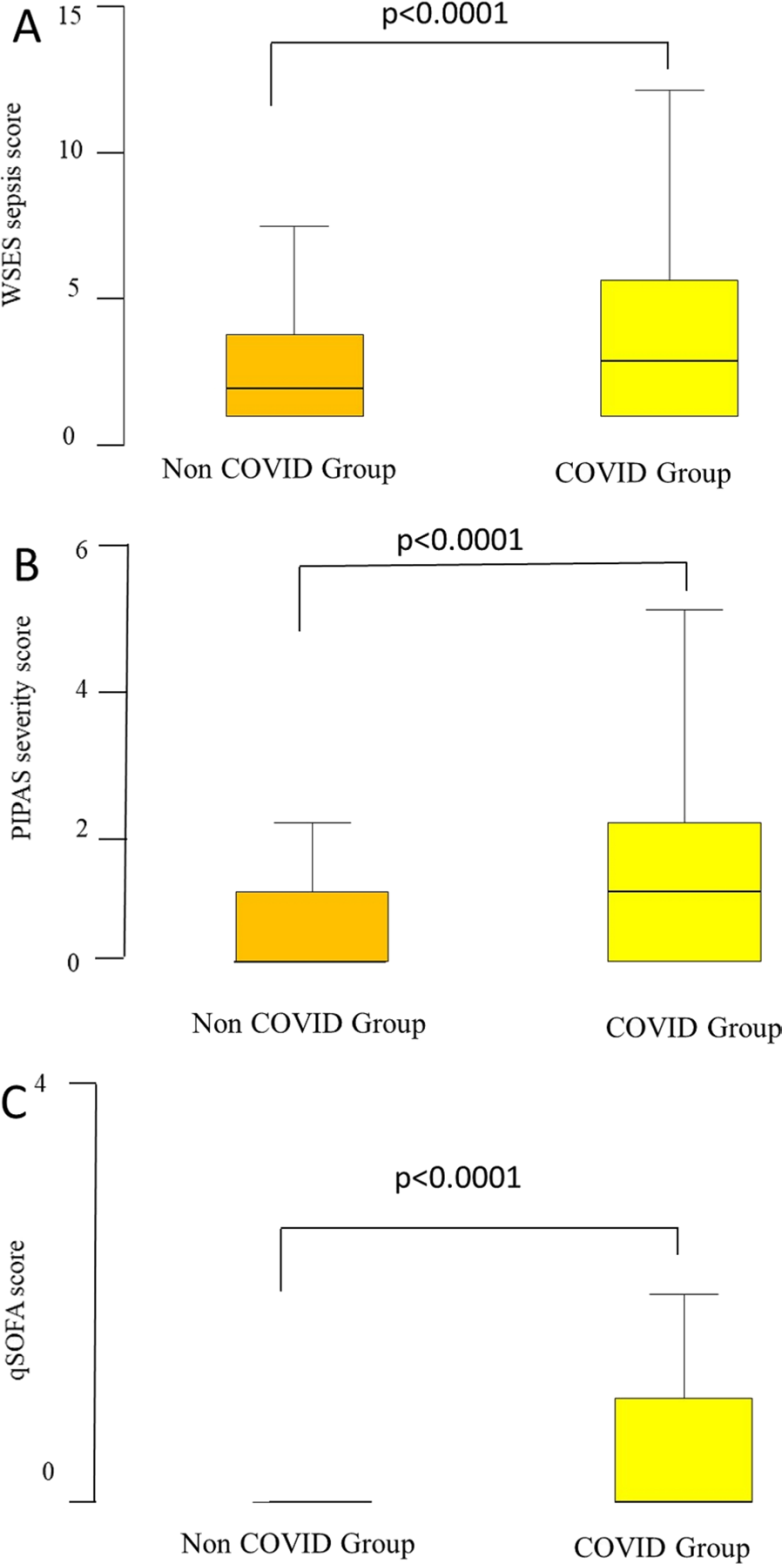
Box-and-whiskers plot of severity markers WSES score (**A**), PIPAS score (**B**), and qSOFA score (**C**), comparing the COVID and the non-COVID patients who were globally treated for acute cholecystitis in 42 countries from 234 centers over the period October 2020–April 2021. The box resembles the 25th percentile and the 75th percentile interquartile range (IQR), while the line within the box resembles the median. *p* value = Mann–Whitney U test

There were 180 patients in the COVID group and 2412 patients in the non-COVID group. Demography of the patients is shown in Table 1. There was no statistical difference of age and gender between the two groups. The rate of concomitant preexisting diseases including cardiovascular diseases (*p* < 0.0001), diabetes (*p* < 0.0001), and severe chronic obstructive airway disease (*p* = 0.005) was significantly higher in the COVID group. Markers of sepsis severity including ARDS (*p* < 0.0001), PIPAS score (*p* < 0.0001), WSES sepsis score (*p* < 0.0001), qSOFA (*p* < 0.0001), and Tokyo classification of severity of AC (*p* < 0.0001) were significantly higher in the COVID group (Table1 and Fig. 1).

**Table 1:** Epidemiological and clinical features of the ChoCO-w population study

Epidemiological and clinical features	Non-COVID 2412	COVID *N* = 180	*p*
Age	61.97 (17.3)	63.93(15.8)	0.21
Gender			012
Male	1268 (52.7%)	84 (46.7%)	
Female	1140 (47.3%)	96 (53.3%)	
Setting of acquisition			0.01
Community based	2027 (89.5%)	143(82.7%)	
Hospital based	239 (10.5%)	30 (17.3%)	
Immunodeficiency	101 (4.2%)	12 (6.7%)	0.13
Malignancy	167 (7%)	13 (7.3%)	0.88
Severe cardiovascular disease	490 (20.4%)	58 (32.2%)	*p* < 0.0001
Diabetes			*p* < 0.0001
No diabetes	1856 (77%)	126 (70%)	
Prediabetes	37 (1.5%)	11 (6.1%)	
History of diabetes	123 (5.1%)	16 (8.9%)	
Diabetes without complications	321 (13.3%)	19 10.6%)	
Diabetes with complication	74 (3.1%)	8 (4.4%)	
Severe CKD	91 (3.8%)	8 (4.5%)	0.55
Severe COPD	155 (6.4%)	22 (12.4%)	0.005
ARDS	24 (1%)	27 (15.2%)	*p* < 0.0001
PIPAS score	0 (0–7)	1 (0–6)	*p* < 0.0001
WSES score	1 (0–15)	2 (0–16)	*p* < 0.0001
qSOFA score	0 (0–5)	0 (0–8)	*p* < 0.0001
Tokyo classification of severity of AC	1.62 (0.66)	1.87 (0.75)	*p* < 0.0001
Patients having complications	282 (11.7%)	57 (32.2%)	*p* < 0.0001
Clavien-Dindo complication score	1 (1–4)	2 (1–4)	*p* < 0.0001
Hospital stay (days)	6.51 (5.6)	13.21 (12.6)	*p* < 0.0001
Mortality	40 (1.7%)	24 (13.4%)	*p* < 0.0001

*AC* acute cholecystitis, *CKD* chronic kidney disease, *COPD* chronic obstructive pulmonary disease, *ARDS* acute respiratory distress syndrome

Patients who had COVID-19 had significantly higher complications (32.2% compared with 11.7%, *p* < 0.0001), longer mean hospital stay (13.21 compared with 6.51 days, *p* < 0.0001), and higher mortality (13.4% compared with 1.7%, *p* < 0.0001) compared with non-COVID patients.

Table 2 shows the clinical presentation of the two groups. COVID patients had significantly more generalized abdominal pain compared with non-COVID patients (20.1% compared with 12.4%, *p* < 0.0001). The COVID group had also significantly higher mean (SD) core body temperature [(37.32 (0.92)°C compared with 36.87 °C (0.81) °C, *p* < 0.0001)], heart rate [(89.7 (14.8) bpm compared with 84.3 (16.6) bpm, *p* < 0.0001], lower systolic blood pressure [(124 (23.4) mmHg compared with 131.5 (23.4) mmHg, *p* < 0.0001], higher respiratory rate [(19.3 (3.73) breaths/min compared with 17.1 (3.25) breaths/min, *p* < 0.0001], lower SpO_2_ [(94% (80–100) compared with 97% (97–100), *p* < 0.0001), and higher incidence of shock (11.2% compared with 3.5%). There was no statistical difference in the modality of preoperative diagnosis between the two groups.

**Table 2:** Clinical findings in COVID and non-COVID patients

Clinical findings	Non-COVID group *n* = 2412	COVID group *n* = 180	*p*
Duration of symptoms (days)	3.66 (7.52)	3.71 (6.85)	0.88
Abdominal findings			0.006
No pain	53 (2.2%)	2 (1.1%)	
Localized pain	1510 (62.8%)	93 (52%)	
Localized pain and rigidity	541 (22.5%)	48 (26.8%)	
Diffuse abdominal pain	299 (12.4%)	36 (20.1%)	
Peritonitis			0.002
Localized	1520 (95.1%)	127 (88.2%)	
Generalized	78 (4.9%)	17 (11.8%)	
Core temperature (°C)	36.87 (0.81)	37.32 (0.92)	*p* < 0.0001
Heart rate (bpm)	84.3 (16.6)	89.7 (14.8)	*p* < 0.0001
Systolic blood pressure (mmHg)	131.5 (23.4)	124 (23.4)	*p* < 0.0001
Respiratory rate (breaths/min)	17.1 (3.25)	19.3 (3.73)	*p* < 0.0001
SpO_2_ (%)	97 (97–100)	94 (80–100)	*p* < 0.0001
Shock	85 (3.5%)	20 (11.2%)	*p* < 0.0001
Preoperative diagnosis			*p* = 0.18
Gallstone cholecystitis	2177 (90.8%)	161 (92%)	
Acalculous cholecystitis	93 (3.9%)	8 (4.6%)	
Biliary pancreatitis	19 (0.8%)	2 (1.1%)	
Gallbladder mucocele	18 (0.8%)	0 (0%)	
CBD stones	85 (3.5%)	3 (1.7%)	
Cholangitis	4 (0.2%)	0 (0%)	
Others	1 (0.04%)	1 (0.6%)	

The COVID-19 group has more generalized abdominal pain (20.1% compared with 12.4%)

*CBD* common bile duct

Table 3 compares the laboratory tests results between the two groups. The mean white blood cell count and CRP were significantly higher in the COVID group [(8156 (8266)/mm^3^ compared with 7501 (18 690)/mm^3^ and 89.44 (98.3) mg/L compared with 80.15 (102.5); *p* = 0.04 and 0.002, respectively]. The most striking significant differences were in the total bilirubin and conjugated bilirubin which were almost doubled in the COVID group [9.07 (19.99) mg/dL compared with 5.38 (26.24) mg/dL and 5.38 (15.89) mg/dL compared with 2.31 (8.14), < 0.0001 in both]. Although there was statistical significance in the mean value of AST and ALT, the difference did not seem to impact on clinical features and outcomes. D-dimer was significantly higher, and arterial lactates were significantly lower in the COVID group [(858.5 (2382) nmol/L compared with 456.8 (1644); *p* = 0.02)] and [(3.52 (12.73) mmol/L compared with 16.96 (79), *p* = 0.03, respectively]. APTT time was significantly longer in the COVID patients [(31.52 (8.94) sec compared with 26.39 (11.54); *p* < 0.0001)].

**Table 3:** Laboratory tests results in COVID and non-COVID patients

Laboratory tests results	Non-COVID group *n* = 2412	COVID group *n* = 180	*p* value
WBC (count/mm^3^)	7 501 (18 690)	8156 (8266)	0.04
Platelets (mm^3^)	119 882 (141 627)	118 550 (130 685)	0.38
C reactive protein (mg/L)	80.15 (102.5)	89.44 (98.35)	0.002
AST U/L value	90.9 (174)	87.7 (108.4)	< 0.0001
ALT U/L value	95.5 (150.3)	94.6 (128.1)	0.001
Total bilirubin (mg/dL)	5.38 (26.24)	9.07 (19.99)	< 0.0001
Conjugated bilirubin (mg/dL)	2.31 (8.14)	5.83 (15.89)	< 0.0001
Indirect bilirubin (mg/dL)	2.43 (15.78)	3.66 (6.39)	0.001
GGT U/L value	141.92 (201.64)	131.5 (156.3)	0.21
Procalcitonin (µg/L)	4.05 (16.52)	4.32(12.8)	0.29
Lactate (mmol/L)	16.96 (79)	3.52 (12.73)	0.03
Fibrinogen (g/L)	307.34 (569.49)	254.1 (322.2)	0.29
D-dimer (nmol/L)	456.8 (1644)	858.5 (2382)	0.02
Prothrombin time (s)	18.1 (20.54)	17.46 (16.29)	0.5
APTT (s)	26.39 (11.54)	31.52 (8.94)	< 0.0001
INR	1.4 (4.13)	1.24 (0.71)	0.017

*WBC* white blood count cells, *AST* aspartate aminotransferase, *ALT* alanine aminotransferase, *GGT* gamma-glutamyl transferase

The difference in mean value of INR in COVID and non-COVID groups [1.24 (SD 4.1) versus 1.4 (SD 0.71)] was not statistically significant (*p* = 0.017).

The management of patients admitted in ED with AC during the COVID-19 pandemic, without distinction of positivity to RT-PCR swab test for COVID infection, is shown in Table 4.

**Table 4:** Management of patients admitted with acute cholecystitis during the COVID-19 pandemic, without distinction of RT-PCR swab test for COVID infection result

Management	Count	%
Endoscopic retrograde cholangiopancreatography (ERCP) ± sphincterotomy and delayed laparoscopic cholecystectomy	183	6
Open intervention in urgent setting + antibiotics	250	8
Conservative approach (antibiotics alone) and delayed laparoscopic cholecystectomy	335	11
Laparoscopic intervention in urgent setting + antibiotics	1474	51
Conservative approach (antibiotics alone)	414	14
Interventional radiology/cholecystostomy/percutaneous drainage of gallbladder	211	7
Conservative approach (antibiotics) + Cholecystectomy/ERCP + delayed laparoscopic cholecystectomy	1	0
Conservative approach with antibiotic treatment-delayed intervention due to patient deterioration-percutaneous cholecystostomy	1	0
	2869	100

Table 5 compares the management between the COVID and non-COVID groups. There was highly significant difference in the surgical management between the two groups, *p* < 0.0001. Laparoscopic total cholecystectomy was performed less frequently in the COVID group (58.1% compared with 76.6%; *p* < 0.0001), while open total cholecystectomy was significantly higher in the COVID group (22.5% compared with 6.7%; *p* < 0.0001). Open total cholecystectomy after conversion was significantly decreased in the COVID group (0.7% compared with 5.4%; *p* < 0.0001). Reoperation was significantly higher in the COVID group (14.6% compared with 2.6%; *p* = 0.011).

**Table 5:** In-hospital management of ChoCO patients: comparison between COVID and non-COVID patients

Management	Non-COVID group *n* = 2412	COVID group *n* = 180	*p*
Primary radiological diagnosis			0.19
Ultrasound	1604 (66.9%)	110 (61.8%)	
CT scan	795 (33.1%)	68 (38.2%)	
Delay in intervention (h)	45.9 (110.1)	63.44 (201.4)	0.89
Surgery			*p* < 0.0001
Laparoscopic total cholecystectomy	1401 (76.6%)	75 (58.1%)	
Laparoscopic total cholecystectomy and intraoperative cholangiography	135 (7.4%)	10 (7.8%)	
Laparoscopic partial cholecystectomy	21 (1.1%)	1 (0.8%)	
Open total cholecystectomy	123 (6.7%)	29 (22.5%)	
Open total cholecystectomy and intraoperative cholangiography	17 (0.9%)	2 (1.6%)	
Open partial cholecystectomy after conversion	18 (1%)	1 (0.8%)	
Open partial cholecystectomy	17 (0.9%)	2 (1.6%)	
Open total cholecystectomy after conversion	98 (5.4%)	9 (0.7%)	
Adequate source control	2206 (94.6%)	158 (93.5%)	0.48
Adequate empirical antibiotics	2317 (97.9%)	169 (95.5%)	0.48
Reoperation	55 (2.6%)	10 (14.6%)	0.011
Strategy for reoperation			0.11
Laparoscopy	16 (23.9)	2 (15.4)	
On demand laparotomy	16 (23.9)	3 (23.1)	
Planned laparotomy	7 (10.4)	5 (38.5)	
Radiological intervention	28 (41.8)	3 (23.1)	
Ventilation	67 (2.8%)	30 (16.8%)	*p* < 0.0001
Ventilation time (days)	5 (6.6)	4.55 (4.1)	0.67
Parenteral nutrition	145 (6.1%)	39 (22.2%)	*p* < 0.0001
Parenteral nutrition time (days)	4.01 (4.78)	6.95 (6.5)	*p* = 0.001

*CT* computer tomography

COVID patients needed significantly more mechanical ventilatory support (16.8% compared with 2.8%, *p* < 0.0001) and parenteral nutrition support (22.2% compared with 6.1%, *p* < 0.0001).

**Fig. 2:**
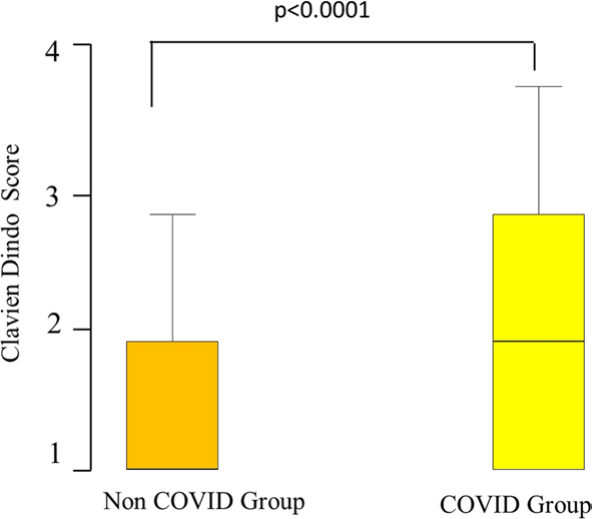
Box-and-whiskers plot of Clavien-Dindo postoperative complication classification comparing the COVID and the non-COVID patients. The box resembles the 25th percentile and the 75th percentile interquartile range (IQR), while the line within the box resembles the median. *p* value = Mann–Whitney U test

The COVID group had significantly higher postoperative complications compared with the non-COVID group (32% compared with 11%, respectively, *p* < 0.0001), including SSI, pulmonary infections, bleeding, and biliary generalized peritonitis (Tables 1, 2, 3, 4, 5 and 6). The Clavien-Dindo complication score was significantly higher in the COVID group [median (range) 2 (1–4) compared with 1 (1–4), *p* < 0.0001, Fig. 2]. The incidence of diffuse biliary peritonitis, biliary fistula, and common bile duct injury was 2.7% (5/180), 1.1% (2/180), and 0.6% (1/180), respectively, in the COVID group.

**Table 6:** Postoperative complications in the COVID and non-COVID-19 patients

Postoperative complications	Non-COVID group *n* = 2412	COVID group *n* = 180
Localized biliary peritonitis	51 (2.1%)	9 (5%)
Pulmonary	44 (1.82%)	12 (6.6%)
Wound infection	39 (1.61%)	15 (8.3%)
Bleeding	32 (1.32%)	5 (2.7%)
Intra-abdominal abscess	26 (1.07%)	1 (0.6%)
Diffuse biliary peritonitis	25 (1.03%)	5 (2.7%)
Biliary fistula	19 (0.8%)	2 (1.1%
Sepsis/septic shock	16 (0.07%)	4 (2.2%)
CBD stones	14 (0.6%)	1 (0.6%)
Gastrointestinal	9 (0.04%)	1 (0.6%)
Cardiac	8 (0.03%)	2 (1.1%)
CBD injury	7 (0.03%)	1 (0.6%)
Fever of unknown source	7 (0.03%)	2 (1.1%)
Bowel perforation	7 (0.03%)	0 (0%)
Localized collection	5 (0.02%)	0 (0%)
Pancreatitis	5 (0.02%)	1 (0.6%)
Renal	3 (0.01%)	1 (0.6%)
Delerium/neurological	3 (0.01%)	3 (1.7%)
Others	14 (0.6%)	1 (0.6%)

The patients may have more than one complication. The percentage of complications are calculated separately from the whole population

*CBD* common bile duct

Mortality rate was 13.4% (24/180) in the COVID group and 1.7% (40/2412) in non-COVID group (*p* < 0.0001).

The detailed postoperative complications of the two groups are shown in Table 6.

**Fig. 3:**
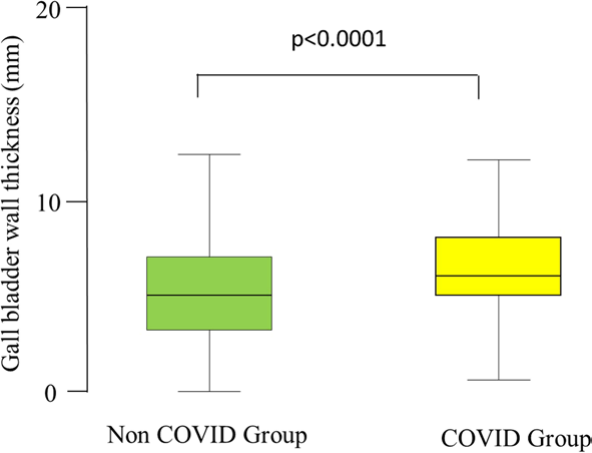
Box-and-whiskers plot of gall bladder wall thickness (mm) in the COVID and the non-COVID patients who had total or partial cholecystectomy. The box resembles the 25th percentile and the 75th percentile interquartile range (IQR), while the line within the box resembles the median. *p* value = Mann–Whitney U test

Table 7 shows the histopathological results in non-COVID and COVID groups. A statistical difference was shown between the two groups (*p* < 0.0001). The incidence of GC was doubled in the COVID group compared with the non-COVID group (40.7% compared with 22.3%). Gallbladder wall was significantly thicker in the COVID group [6.32 (2.44) mm compared with 5.4 (3.45) mm; *p* < 0.0001] (Fig. 3).

**Table 7:** Histopathologic findings in COVID and non-COVID patients

Histopathology	Non-COVID group	COVID group
Acute cholecystitis	899 (47.8%)	58 (43%)
Chronic cholecystitis	489 (26%)	18 (13.3%)
Cholecystitis with necrosis/gangrene	419 (22.3%)	55 (40.7%)
Acute on chronic cholecystitis	46 (2.4%)	1 (0.7%)
Perforated cholecystitis/abscess formation	11(0.6%)	2 (0.15%)
Malignancy	10 (0.5%)	1 (0.7%)
Hydrocele	2 (0.11%)	0 (0%)
Adenosis	2 (0.11%)	0 (0%)
Normal	1 (0.05%)	0 (0%)
Total	1879 (100%)	135 (100%)

## Discussion

To our knowledge, the ChoCO-W study is the largest global prospective study comparing COVID and non-COVID patients admitted with the diagnosis of AC. Recently, the CHOLECOVID study was published [18]. The methodology and aim of this study are different from ours. The CHOLECOVID study retrospectively compared the management of AC during the COVID pandemic with the pre-pandemic period. Instead we prospectively compared the characteristics and outcomes of patients who tested positive for SARS-CoV-2 during the episode of AC with those who did not.

Furthermore, in the ChoCO-W study recruitment period, Delta SARS-CoV-2 variant (B.1.617.2) was the most circulating virus and it was associated with higher transmissibility compared with wild-type SARS-CoV-2 and decreased vaccine effectiveness with higher incidence of secondary attack than the Alpha variant (B.1.1.7) [ https://assets.publishing.service.gov.uk/government/uploads/system/uploads/attachment_data/file/992983/21_May_2021_Risk_assessment_for_SARS-CoV-2_variant_VOC-21APR-02__B.1.617.2_.pdf].

During this first part of COVID-19 pandemic, health facilities were collapsing and people was recommended to stay home to limit human contact and the spreading of the virus.

The access to emergency departments was limited to patients with respiratory failure and acute abdomen with sepsis and septic shock.

Operating theaters were converted in ICUs and healthcare staff reallocated to manage patients with ventilatory support; consequently, access to OR was restricted to surgical patients non-eligible for NOM or after medical treatment failure in keeping the adequate personal protective equipment availability and decreasing the in-hospital circulation of the virus.

RT-PCR swab test result was mandatory to be admitted in OR.

The reported mortality of patients having GC is high mortality rate, and it increases in elderly and diabetic patients [19, 20].

Our study showed that COVID-19 patients with AC have an increased risk of presenting GC with higher postoperative complications and mortality rate.

This can be attributed to the associated comorbidity and frailty of COVID-19 patients, needing more frequently ventilatory mechanical support and parenteral nutrition and presenting with higher sepsis scores.

However, the environment may have contributed to enroll the most comorbid and severe patients in our study and probably to increase delay in surgical management (delay to ED admission + delay to OR admission) with negative outcomes and longer hospital stay.

Our data did not confirm an higher delay to surgical management; in fact, the mean (hours) delay from admission to surgical management was 63.44 (SD 201.4) and 45.9 (SD 110.1), respectively, for COVID and non-COVID groups (*p* = 0.89).

COVID patients had lower arterial lactate values compared to non-COVID patients [(3.52 (12.73) mmol/L compared with 16.96 (79), *P* = 0.03, respectively].

This is an unexpected result, since COVID patients had higher sepsis scores and signs of shock compared with non-COVID patients.

Carpenè et al. [20] reviewed 19 studies about hyperlactatemia and severe COVID disease, with 6459 patients included. They reported that COVID-19 patients with worse outcome have usually higher lactate values than those with better outcome, but most COVID-19 patients did not show hyperlactatemia, even if critically ill.

The association between blood lactate values and clinical outcome remains unclear in patients with SARS-CoV-2 infection. COVID-19 pathogenesis is multifactorial, in some way independent from severe ischemia and hyperlactatemia; in fact, patients with COVID-19 pneumonia or ARDS are reported with lower blood lactate values compared to those with non-COVID-19 pneumonia or ARDS of different etiologies [21].

Moreover, hyperlactatemia in COVID patients could be induced by medications such as metformin, propofol, acetaminophen [22–24], and catecholamines.

Iepsen et al. [25] reviewed the literature to assess if pathophysiology of lactate metabolism in sepsis and COVID patients is different from non-COVID septic patients. Evidence supports that elevated blood lactate value is strongly associated with mortality in septic patients. Lactatemia value seems unrelated to tissue hypoxia but likely reflects mitochondrial dysfunction and high adrenergic stimulation. Patients with severe COVID-19 exhibit near-normal blood lactate, indicating preserved mitochondrial function, despite a systemic hyperinflammatory state similar to sepsis.[25].

There is a need for further studies to assess this outcome. Nevertheless, serum lactate values monitoring in COVID patients may be useful for early identification of higher risk COVID-19 illness progression, but hyperlactatemia in severe COVID patients may not be present [22].

Our COVID-19 patients had higher total serum bilirubin, mostly conjugated, supporting the hypothesis that SARS-CoV-2 has a tropism for hepatic cells [26–28]. Several mechanisms were proposed to explain SARS-CoV-2 hepatic injury in critically ill patients including hypoxic hepatitis due to shock, high levels of positive end-expiratory pressure leading to hepatic congestion, and medications such as lopinavir/ritonavir. Most of our patients were not supported by mechanical ventilation. Despite that, they had abnormal liver functions most likely because of the hepatic ACE2 receptors which interact with SARS-CoV-2 causing direct cytopathic effects [26]. Patients with abnormal liver functions have at higher risk of progressing to severe COVID disease [28].

The COVID group showed a longer aPTT time and lower INR value compared with the non-COVID group in our study, and this would suggest intrinsic clotting factor deficiency.

This evidence supports published data about coagulability disorders of COVID-19 patients, characterized by significantly elevated D-dimer and fibrinogen (hyper-coagulability), mild thrombocytopenia and a mildly prolonged PT/aPTT (hypo-coagulability), based mainly on immunothrombosis mechanism which is triggered by hyperinflammatory response and diffuse endotheliopathy. This endothelial derangement most often manifests as an early hypercoagulable state with high risk of venous and arterial thromboembolic events and then results in a hemostatic derangement known as fibrinolytic shutdown [29, 30].

Elevated D-dimer levels in COVID patients are consistently reported, whereas their gradual increase during disease course is particularly associated with disease progression. PT and aPTT prolongation and fibrin degradation products’ increase with severe thrombocytopenia are correlated with life-threatening disseminated intravascular coagulation (DIC) [31–33].

Tang et al. [34] reported early that high D-dimer and fibrin degradation product (FDP) levels are risk factors for DIC and death in severe COVID-19 patients. Their study showed a significantly higher D-dimer and FDP levels and longer PT and aPTT in non-survivors compared to survivors on admission (*p* < 0.05) [34].

Venous or arterial thrombotic complications are reported in one-third of ICU COVID-19 patients despite pharmacological thrombo-prophylaxis [29, 35].

COVID-19 disease is associated with hypo-fibrinolysis as shown by thromboelastogram assays, but due to the costs of this laboratory exam, we did not collected sufficient data for analysis. Elevated D-dimer suggests hyper-fibrinolysis. This increases the risk of thrombotic events and renal failure which increases mortality rate [29]. SARS-CoV-2 may lead to direct endothelial injury and increased levels of pro-inflammatory cytokines (such as tumor necrosis factor-α, interleukin-1, and interleukin-6 leading to a cytokine storm). This has been associated with micro- and macrovascular thrombosis and organ failure [31]. The WSES was the first society to recommend early administration of prophylactic anticoagulation with LMWH in COVID-19 surgical patients to reduce the risk of thromboembolism [36]. The CORIST (Italian retrospective multicentric observational) study [37], which enrolled 2574 patients, showed that in-hospital heparin treatment was associated with a lower mortality, particularly in severely ill COVID-19 patients and in those with strong coagulation activation.

The International Society of Thrombosis and Haemostasis recommended measuring D-dimers, prothrombin time, and platelet count in all patients who present with COVID-19 infection in stratifying patients who may need admission and close monitoring or not [38].

The COVID-induced micro-angiopathy and hyper-coagulability could be correlated with the high incidence of GC in COVID-19 patients, but the ChoCO-W study cannot confirm this. Nevertheless, our study showed that the incidence of GC was doubled in COVID patients group compared with non-COVID (40.7% compared with 22.3%; *p* > 0.0001) and gallbladder wall was significantly thicker in COVID patients.

This was previously considered as a risk factor for “difficult gallbladder” surgery associated with higher conversion rate. In contrast, our data have shown that laparoscopic cholecystectomy, performed in 58% (75/180) of COVID-19 patients, is a safe and reproducible procedure in expert hands with a conversion rate of only 0.7% (compared with 5.4% in non-COVID group; *p* < 0.0001), that is, lower than the reported conversion rates for GC (ranging from 18 to 25%) [39, 40].

Open total cholecystectomy in our study was performed in 22.5% of the COVID-19 patients compared with 6.7% of the non-COVID patients. This is probably due to the hemodynamic instability and respiratory failure of COVID patients enrolled in our study: Nobody will perform a laparoscopic approach in hemodynamic unstable patients and in surgical patients presenting hypoxic respiratory failure.

Furthermore, several international surgical societies recommended against performing laparoscopic cholecystectomy because of the potential risk of SARS-CoV-2 transmission correlated with surgical smoke and artificial pneumoperitoneum: This may have leaded surgeons to reduce the use of laparoscopy in COVID patients.

To our knowledge, there are no data confirming increased risk of contamination among healthcare providers during laparoscopy and laparoscopic cholecystectomy is the golden standard treatment for cholecystitis in all patients [2].

However, in our study (laparoscopic and open) cholecystectomy showed a slightly higher rate of biliary leakage in COVID patients (1.1%) compared with non-COVID patients (0.8%) although not statistically significant. These data are slightly higher than biliary leakage rates reported in the literature [41–43].

Subtotal cholecystectomy, which was reported to be useful in the management of difficult gallbladders [44], was performed laparoscopically in 1.1% of the non-COVID patients and 0.8% of the COVID patients in our study.

Open partial cholecystectomy after conversion was performed in 1% of the non-COVID patients and 0.8% of the COVID patients. A second surgical exploration was required for 5.5% of the COVID patients compared with 2.6% of the non-COVID patients. COVID-19 patients had statistically higher postoperative complications, higher mean hospital stay (13.21 days compared with 6.51 days), and higher mortality (13.4% compared to 5.4%), similar to other studies [45].

The COVID group had more SSI, pulmonary infections, postoperative bleeding, and diffuse biliary peritonitis, compared with the non-COVID group.

This evidence supports the recommendation to delay surgical management in COVID patients having AC, according to their comorbidities, frailty, severity of pneumonia, and surgical risk in order to decrease postoperative complications and mortality rate, when it is possible [36, 46].

Several early retrospective studies reported an increased use of NOM and percutaneous cholecystostomy (PC) in treating both COVID and non-COVID patients presenting with AC during the early phase of the pandemic because of concerns about the safety of laparoscopy, artificial pneumoperitoneum, and biological fluids in spreading the virus in the operating rooms, and because of limited access to the operating rooms. This approach was associated with increased hospital stay, NOM failure, and increased in-hospital COVID infection [10, 47, 48].

In our study, laparoscopic cholecystectomy was performed in 1474/2869 (51%); NOM including antibiotics alone was used in 14% (414/2869) of COVID and non-COVID patients. The overall open cholecystectomy rate was 8% (250/2869), and PC was performed for 7% of (COVID and non-COVID) patients (211/2869).

To our knowledge, this confirms that PC is not an alternative to laparoscopic cholecystectomy in stable, non-critically ill patients, when an early and safe laparoscopic cholecystectomy can be performed. PC can be considered as a bridge to surgery in unstable, high risk, and unfit patients for surgery [49].

### Strengths and limitations of the study

We enrolled prospectively all the COVID and non-COVID patients admitted with acute cholecystitis in ED in a 6-month period from October 2020 to April 2021. In this first period of Delta variant (higher virulence compared with wild-type SARS-CoV-2) COVID pandemic, only comorbid patients with acute abdominal pain and signs of sepsis were addressed and admitted to ED, overcrowded by severe COVID patients requiring ventilatory support and admission in ICU, because of governments lockdown and limited resources (beds, personal protective equipment, ventilators, operating rooms, and healthcare personnel).

Furthermore, several emergency surgeons opted for open cholecystectomy, when a safe laparoscopy was not possible in limit the spreading of the virus in OR.

We have to acknowledge that the COVID cohort is small and sicker and that the follow-up period of 1 month is short.

The long-term follow-up especially in those who had COVID-19 would be of interest in a future study.

However, this study has a wholistic approach looking for the global outcome without having a specific management protocol despite the major variation between the different countries. This is useful for the generalizability of the study.

To our knowledge, the ChoCO-W study is the first global study about AC comparing COVID and non-COVID patients during the ongoing pandemic.

## Conclusions

The incidence of gangrenous cholecystitis is higher in COVID patients, and it is associated with high-grade Clavien-Dindo postoperative complications, higher length of hospital stay and higher mortality.

When it is possible, it is recommended to delay the surgical treatment in COVID-19 patients to decrease morbidity and mortality rates. Laparoscopic cholecystectomy is the golden standard treatment for acute cholecystitis in all patients. In expert hands, laparoscopic cholecystectomy is a safe and reproducible surgical procedure for acute cholecystitis, without significant increase in biliary leakage rate in COVID and non-COVID patients.

The rate of open cholecystectomy is higher in COVID patients compared with non-COVID patients, without statistically significant difference. To our knowledge, the laparoscopic approach is not associated with an increased biological risk of SARS-CoV-2 transmission in operating room, in presence of adequate protective personal equipment, protocols and skilled staff to manage COVID patients. Gangrenous cholecystitis is not an absolute contraindication to the laparoscopic approach in COVID and non-COVID patients. 

## Data Availability

Not applicable

## Abbreviations

AC: Acute cholecystitis
GC: Gangrenous cholecystitis
LC: Laparoscopic cholecystectomy
NOM: Non-operative management
PC: Percutaneous transhepatic cholecystostomy
WSES: World Society of Emergency Surgery
CKD: Chronic kidney disease
COPD: Chronic obstructive pulmonary disease
ARDS: Acute respiratory distress syndrome
CBD: Common bile duct
SSI: Surgical site infection
LOS: Length of hospital stay
ED: Emergency department
PT: Prothrombin time
APTT: Activated partial thromboplastin time
